# Evolutionary loss of inflammasomes in the Carnivora and implications for the carriage of zoonotic infections

**DOI:** 10.1016/j.celrep.2021.109614

**Published:** 2021-08-24

**Authors:** Zsofi Digby, Panagiotis Tourlomousis, James Rooney, Joseph P. Boyle, Betsaida Bibo-Verdugo, Robert J. Pickering, Steven J. Webster, Thomas P. Monie, Lee J. Hopkins, Nobuhiko Kayagaki, Guy S. Salvesen, Soren Warming, Lucy Weinert, Clare E. Bryant

**Affiliations:** 1 University of Cambridge, Department of Veterinary Medicine, Cambridge CB30ES, UK; 2 Sanford Burnham Prebys Medical Discovery Institute, 10901 North Torrey Pines, La Jolla, CA 92037, USA; 3 Department of Physiological Chemistry, Genentech, South San Francisco, CA 94080, USA; 4 Department of Molecular Biology, Genentech, South San Francisco, CA 94080, USA; 5 University of Cambridge, School of Clinical Medicine, Box 111, Cambridge Biomedical Campus, Cambridge CB2 0SP, UK

**Keywords:** NLRP3, NLRC4, inflammasome, caspase 1, caspase 11, caspase 4, Carnivora

## Abstract

Zoonotic pathogens, such as COVID-19, reside in animal hosts before jumping species to infect humans. The Carnivora, like mink, carry many zoonoses, yet how diversity in host immune genes across species affect pathogen carriage is poorly understood. Here, we describe a progressive evolutionary downregulation of pathogen-sensing inflammasome pathways in Carnivora. This includes the loss of nucleotide-oligomerization domain leucine-rich repeat receptors (NLRs), acquisition of a unique caspase-1/-4 effector fusion protein that processes gasdermin D pore formation without inducing rapid lytic cell death, and the formation of a caspase-8 containing inflammasome that inefficiently processes interleukin-1β. Inflammasomes regulate gut immunity, but the carnivorous diet has antimicrobial properties that could compensate for the loss of these immune pathways. We speculate that the consequences of systemic inflammasome downregulation, however, can impair host sensing of specific pathogens such that they can reside undetected in the Carnivora.

## Highlights

• Carnivorans lack key NLRs and express a unique caspase-1/-4 hybrid protein
• This protein is defective in mediating activation of common inflammasome pathways
• What little activity occurs is driven by caspase-8, rather than caspase-1/-4

## Graphical abstract

**Figure:**
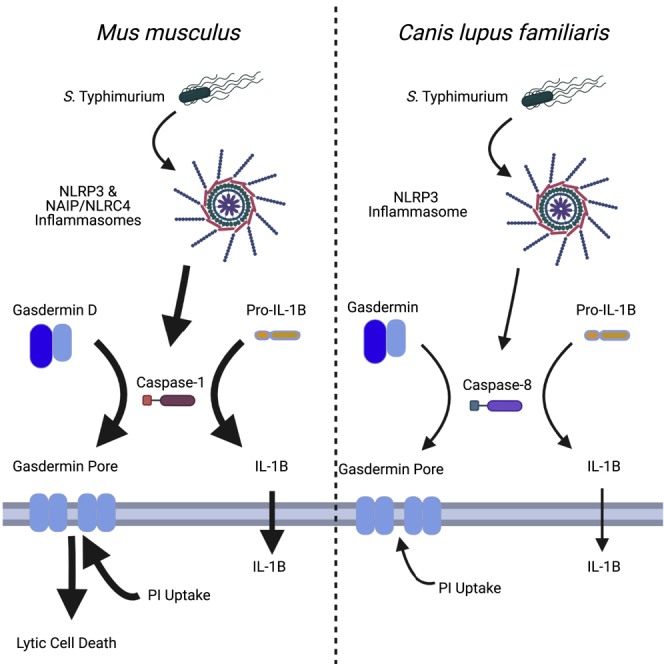

## Teaser

Species of the order Carnivora have evolutionarily acquired the expression of a unique caspase-1/-4 hybrid protein. Digby et al. show that this protein is a poor mediator of NLRP3- and caspase-4-dependent inflammasome activation. This downregulation in inflammasome pathways could impair pathogen detection and facilitate transmission of zoonotic infections.

## Introduction

Viral and bacterial zoonotic pathogens, such as coronavirus disease 2019 (COVID-19) and *Salmonella* species, can infect animal hosts in an asymptomatic or symptomatic manner, which may facilitate the transmission to humans. Pathogen genomics have yielded important discoveries about the diversity of different microorganisms in the context of disease (Weinert et al., 2015). Comparative biology of animal immune systems and their links to infection susceptibility are less well understood. This is partly due to a lack of tools, for example, antibodies or other resources that make immune studies tractable, but the use of CRISPR-Cas9 gene editing is a universal technique that can be applied to cells from many animals. Approximately 49% of all carnivore species (e.g., mink, dogs), the highest proportion of any mammal order including bats, carry one or more unique zoonotic pathogens (Han et al., 2016). Whether this is because Carnivora are a large group of animals harboring many pathogens, so they carry proportionally more zoonoses (Mollentze and Streicker, 2020), or due to other factors such as differences in the immune system remains to be determined.

Inflammasomes are of central importance in host protection against viral and bacterial diseases and drive inflammation to control infections in humans and mice (Broz and Dixit, 2016). Canonical inflammasomes are multi-protein complexes composed of a pathogen-recognition receptor, such as a nucleotide-oligomerization domain leucine-rich repeat receptor (NLR; NLRP1, NLRP3 and NLRC4), pyrin or absent-in-melanoma 2 receptor (AIM2), an adaptor (apoptosis-associated speck-like protein [ASC]), and an effector protein (caspase-1; CASP1). The role of this pro-inflammatory protein complex is to process the immature cytokines pro-interleukin-1β (IL-1β) and pro-IL-18 into their mature, more active forms and to cleave the lytic pyroptotic cell death effector gasdermin D to its pore forming N-terminal fragment (Broz and Dixit, 2016). Non-canonical inflammasomes can also be formed by cytosolic delivery of the bacterial toxin lipopolysaccharide (LPS), which activates caspase-11 in mice or caspase-4 and -5 in humans to cleave gasdermin D, which in turn activates NLRP3 (Broz and Dixit, 2016; Broz et al., 2020; Lieberman et al., 2019). There is wide species diversity in AIM-2 like receptors (ALRs), with AIM2 being non-functional in many species. Evolutionary analysis reveals considerable plasticity in mammalian ALR genes, with no single ALR gene preserved among all mammals. Instead, the ALR genes have undergone extensive, species-specific diversification, suggesting that evolutionary pressures may have shaped ALR sequences and functions throughout the mammals (Brunette et al., 2012).

Here, by comparing the distribution and evolution of inflammasome and cell death genes across the order Carnivora, we find a profound compromise in inflammasome functionality, caspase-dependent lytic cell death pathways, and a critical loss of NLR genes. A caspase-1/caspase-4 fusion protein found in all Carnivora, despite being functionally capable of processing substrates *in vitro*, is inactive in cells from a model carnivore (dog). Caspase-8, which is conserved in the Carnivora, processes delayed pro-IL-1β and upregulates the expression of this protein. This compromised inflammasome activity, coupled to the absence of the necroptotic effector mixed-lineage kinase domain-like pseudokinase (MLKL) (Dondelinger et al., 2016), suggests that the order Carnivora are immunologically challenged, particularly in gut mucosal immunity, but ecology studies suggest that a high-protein diet, such as that consumed by carnivores, has antimicrobial properties. This may explain why these innate immune pathways have been lost in the Carnivora, but the consequences for the carriage of zoonotic pathogens, particularly in organs other than the gut, may be detrimental.

## Results

### The Carnivora caspase-1/-4 fusion has limited activity within dog cells compared to the recombinant protein or when an equivalent caspase-1/-11 fusion protein is expressed within mouse cells

**Figure 1:**
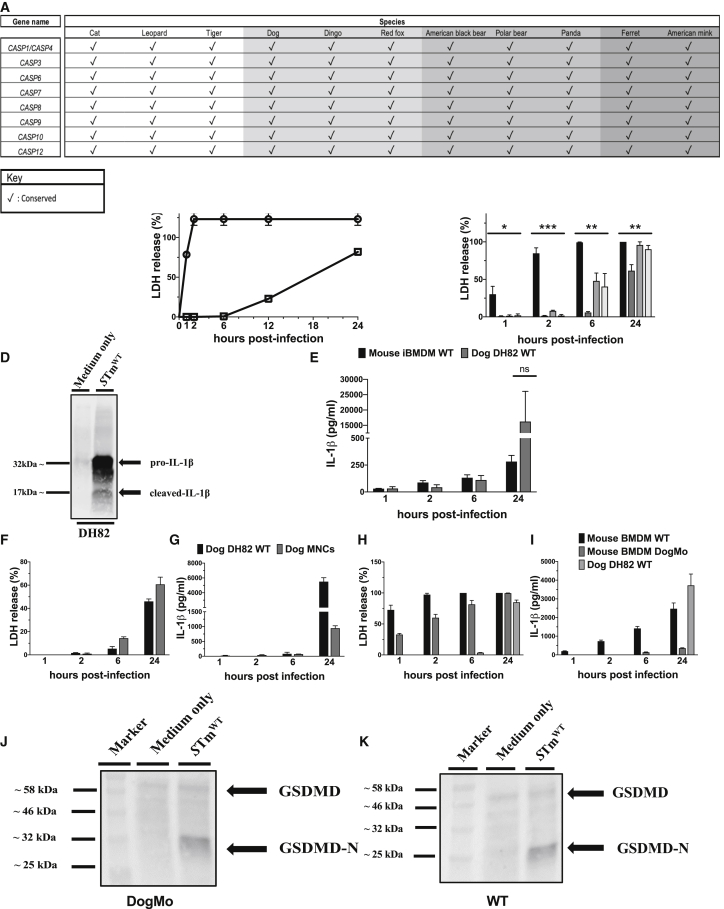
The activity of the Carnivora caspase-1/-4 fusion protein is markedly restricted in cells infected with *S*. Typhimurium (A) Representative table showing the evolutionary conservation of the inflammatory hybrid caspase-1/-4 and apoptotic caspases in species belonging to order Carnivora. (B) Mouse WT iBMDM and dog WT DH82 cells were infected with *S*. Typhimurium MOI of 10, and the amount of lactate dehydrogenase (LDH) released in the supernatant was measured via colorimetric assay over time. (C and E) Mouse WT, Nlrc4^−/−^, *Casp1*^*−/−*^/*11*^*−/−*^ iBMDM, and dog WT DH82 cells were infected with *S*. Typhimurium MOI of 10, and the amounts of LDH and IL-1β released in the supernatant were measured over time via colorimetric assay and ELISA, respectively. (D) Lysates from dog WT DH82 cells infected with *S*. Typhimurium MOI of 1 for 24 h in the supernatant were blotted against dog-specific IL-1β alongside non-infected controls (medium only). (F and G) Primary dog MNCs and dog WT DH82 cells were infected with *S*. Typhimurium MOI of 10, and the amounts of LDH and IL-1β released in the supernatant were measured over time, as in (C) and (E). (H and I) Mouse primary WT and DogMo BMDM together with dog WT DH82 cells were infected with *S*. Typhimurium MOI of 10, and the amounts of LDH and IL-1β in the supernatant were measured over time, as in (C) and (E). (J and K) Mouse primary WT and DogMo BMDMs were infected with *S*. Typhimurium at an MOI of 10 for 6 h, and GSDMD cleavage was determined by western blot analysis of cell lysates using anti-mouse GSDMD antibody. Data are shown as means ± SEMs in (B), (C), and (E)–(I). Data are pooled from 3 independent experiments in (B), (C), and (E), from 1 representative of 3 independent experiments in (D), from 1 representative of 2 independent experiments in (H)–(K), and from 1 single experiment in (F) and (G) for which cells were pooled from 4–6 dogs (Table S1). Statistical significance in (C) and (E) was calculated by a 1-way ANOVA for each time point individually, followed by Tukey’s multiple comparison test; ns, not significant; ^∗^p < 0.05, ^∗∗^p < 0.01, ^∗∗∗^p < 0.001. See also Figures S1 and S2 and Table S1.

Carnivora, such as dogs, cats, and mink, through their close proximity with humans, can be susceptible to human pathogens. There are, however, marked differences in Carnivora inflammasome effector caspases compared to humans and mice (Figure S1A). A unique caspase-1/-4 fusion protein is present in all cats and dogs (Figure S1B) (Eckhart et al., 2008). This protein has the equivalent of the CARD1 domain of caspase-1, which should render it unable to respond to cytosolic LPS, while its catalytic domain is most closely related to that of mouse caspase-11 (caspase-4 or -5 in humans), being only distantly related to caspase-1 in terms of sequence identity (Figure S1B). This caspase-1/-4 fusion is conserved across all Carnivora (Figure 1A) and constitutively expressed in dog cells as shown by mass spectrometry analysis (Figures S1D and S1E). The absence of individual caspase-1 and caspase-4/-5/-11 genes in Carnivora suggests that there will be differences in how inflammasomes function in species of this order. The structure of the caspase-1/-4 fusion (Figure S1C) suggests it should be able to process gasdermin D in response to canonical inflammasome stimulation, but have a limited/no capacity to process IL-1β and IL-18. Analysis of dog gasdermin D shows that both domains are conserved and, although the linker region is more divergent, the aspartate cleavage site is present (Wang et al., 2020), consistent with full functionality of this protein in the Carnivora.

To characterize inflammasome functionality in Carnivora, we compared the kinetics and magnitude of cell death between immortalized bone marrow-derived macrophages (iBMDM) from wild-type (WT) mice and the DH82 dog macrophage-like cell line (used as a model for the Carnivora). Cells were infected with *Salmonella enterica* serovar Typhimurium (*S.* Typhimurium), which activates NLRC4 and NLRP3 canonical inflammasome formation (Man et al., 2014). All of the mouse cells lysed within the first 2 h post-infection, while dog cells were more resistant and survived well beyond 12 h (Figure 1B). Mouse *casp1*^*−/−*^*/11*^*−/−*^ cells, as expected, were also resistant to rapid cell death, but, unlike dog cells, started to lyse at 6 h post-infection (Figure 1C). Interestingly, dog cells showed clear processing of pro-IL-1β (Figure 1D) accompanied by the release of large amounts of IL-1β at 24 h post-infection (Figure 1E). Experiments in primary monocyte-derived macrophages (MNCs) isolated from dog peripheral blood mononuclear cells infected with *S*. Typhimurium for 24 h also induced IL-1β production with delayed lytic cell death (Figures 1F and 1G). This is unexpected because, based on the structure, this hybrid protein was predicted to process gasdermin D to lyse cells, but not cleave IL-1β in response to canonical inflammasome activity (Kayagaki et al., 2011).

**Figure 2:**
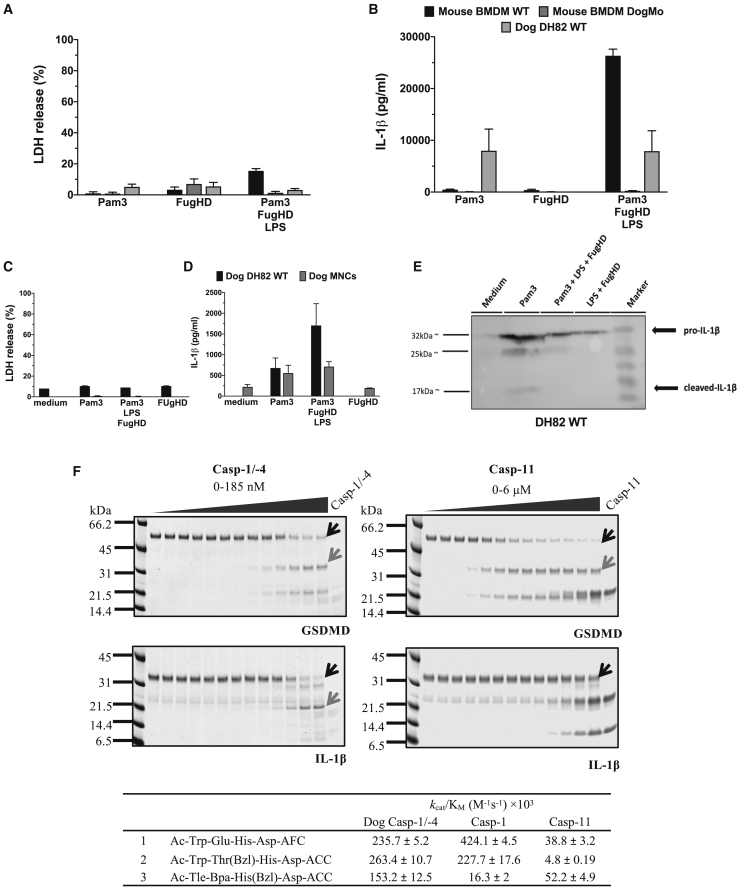
The Carnivora caspase-1/-4 fusion protein induces substrate cleavage *in vitro*, but fails to activate the non-canonical inflammasome pathway *in vivo* (A and B) Mouse primary WT and DogMo BMDM together with dog WT DH82 cells were primed with Pam3CSK4 (10 μg/mL for 4 h) and then transfected with LPS (5 μg/mL for 16 h) using Fugene HD. The amounts of LDH and IL-1β released in the supernatant were measured over time via colorimetric assay and ELISA, respectively. (C and D) Primary dog MNCs and dog WT DH82 cells were stimulated and analyzed as in (A) and (B). (E) Lysates from dog WT DH82 cells primed and stimulated as in (A) and (B) were blotted against dog-specific IL-1β alongside the appropriate controls. (F) Upper: *in vitro* protein cleavage. Recombinant mouse gasdermin D and pro-IL-1β were submitted to cleavage with a dilution series of dog caspase-1/-4 or mouse caspase-11, incubated for 30 min, and followed with SDS-PAGE analysis of cleavage products. Black arrows indicate bands corresponding to intact substrate, and gray arrows correspond to the biologically active form of the protein originated by caspase cleavage. Lower: catalytic efficiency represented as kcat/KM (M-1 s-1) values for peptidyl fluorogenic substrates (1) general inflammatory caspase substrate, (2) caspase-1 selective substrate, or (3) caspase-11 selective substrate. Average of 3 determinations ± SDs. Data are shown as means ± SEMs in (A)–(D). Data are pooled from 2 independent experiments in (A) and (B), from 1 representative of 2 independent experiments in (E), and from 1 single experiment in (C) and (D) for which cells were pooled from 4–6 dogs. See also Table S1 and Figure S3.

To test this predicted activity, a novel mouse strain that carries a caspase-1/-11 fusion, equivalent to the one found in the Carnivora, was generated. Caspase-1 and caspase-11 are so close to each other in the mouse genome (Kayagaki et al., 2011) that it is possible to delete the catalytic domain of caspase-1 and the N-terminal caspase recruitment domains (CARDs) of caspase-11 to make a mouse fusion protein equivalent to that found in the Carnivora. We therefore used a novel approach of CRISPR-Cas9 gene deletion to generate a mouse that expresses a fusion protein consisting of the caspase-1 CARD1 and the caspase-11 catalytic domain (DogMo) and confirmed expression via western blot analysis (Figures S2C and S2D). BMDMs from DogMo infected with *S*. Typhimurium, as expected, showed canonical inflammasome-driven cell lysis and gasdermin D processing, but no IL-1β production (Figures 1H–1K). Cell lysis in DogMo BMDMs was, interestingly, reduced very early during infection when compared to infected WT BMDM (Figure 1H). DogMo cells also showed no cell lysis or IL-1β production in response to non-canonical inflammasome activation induced by cytosolic LPS (Figures 2A and 2B). Similarly, DH82 dog cells and primary dog mononuclear cells showed no cell lysis (Figures 2C and S3A), and the amount of IL-1β produced was the same whether cells were primed with the Toll-like receptor 2 (TLR2) ligand Pam3CSK4 alone (as a control) or after priming with Pam3CSK4 and then transfected with LPS (Figures 2B, 2D, and S3B). This suggests that this cytokine was induced by priming rather than non-canonical inflammasome activation, which was confirmed by western blot analysis (Figure 2E). To determine whether the enzymatic properties of the caspase-1/-4 hybrid could account for IL-1β processing in the absence of lytic cell death by inflammasomes in dog cells, we expressed the catalytic domain of this protein and tested its ability to process substrates *in vitro* (Figure 2F). Caspase-1/-4 processed both gasdermin D and IL-1β to their biologically active forms *in vitro*. Caspase-11, as expected, cleaved gasdermin D but not IL-1β (Figure 2F). We also used synthetic peptidyl substrates optimized, based on specificity screens, to improve selectivity for caspase-1 or caspase-11 (Ramirez et al., 2018). Dog caspase-1/-4 cleaved these synthetic substrates at superior rates compared to caspase-11 and cleaved the caspase-1 optimum substrate at rates comparable to caspase-1 (Figure 2F). These data reveal that the substrate specificity of dog caspase-1/-4 resembles that of caspase-1 more closely than caspase-11 *in vitro*. This suggests that the defective canonical and non-canonical inflammasome responses in dog cells are not caused by an intrinsic loss of enzymatic activity of the caspase-1/-4 fusion protein, but most likely because of an alternative regulatory mechanism.

### The inflammasome gene repertoire and the NLRP3 functionality, in particular, are severely compromised in the Carnivora

**Figure 3:**
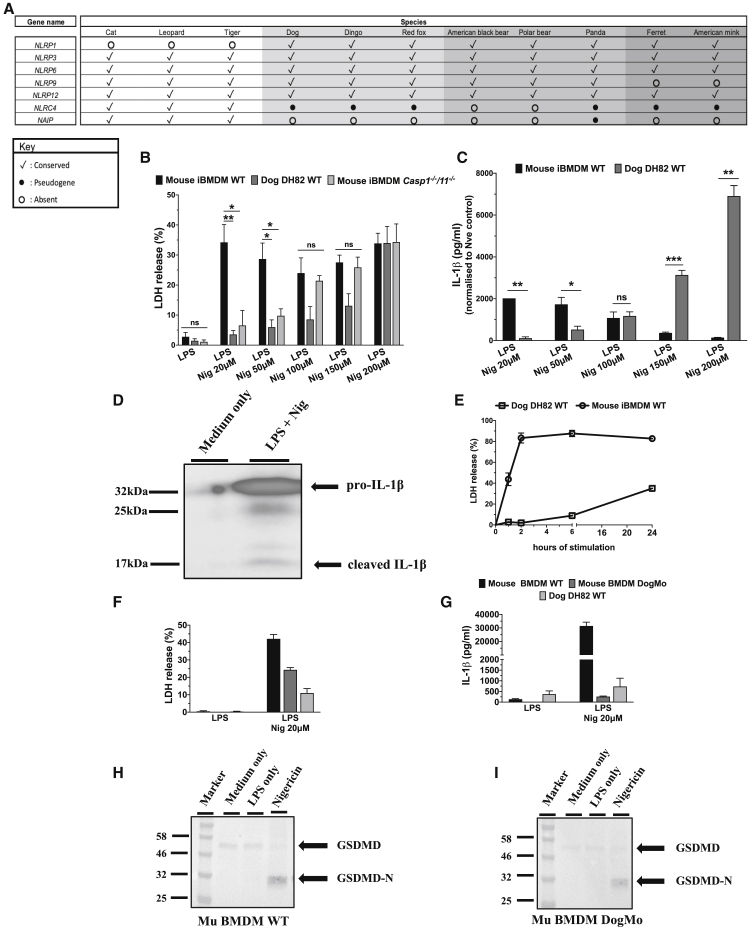
The NLR gene repertoire and NLRP3-dependent inflammasome pathway are severely compromised in the Carnivora (A) Representative table showing the evolutionary conservation of pattern recognition receptors (PRRs) in species belonging to order Carnivora. (B) Mouse WT iBMDM, mouse *Casp1*^*−/−*^/*11*^*−/−*^ iBMDM, and dog WT DH82 cells were primed with LPS (200 ng/mL for 3 h) and then stimulated with nigericin (20–200 μM for 1 h). The amount of LDH released in the supernatant was measured over time via a colorimetric assay. (C) The same experiment as in (B); the amount of IL-1β in the supernatant was measured for mouse WT and dog WT DH82 cells only via ELISA. (D) Dog WT DH82 cells were primed with LPS (200 ng/mL for 3 h) and then stimulated with nigericin (200 μM for 1 h). Total protein was precipitated from the supernatant, and IL-1β cleavage was assessed by western blot analysis. (E) Mouse WT iBMDM and dog WT DH82 cells were primed with LPS (200 ng/mL for 3 h) and then stimulated with nigericin (10 μM for 24 h). The amount of LDH released in the supernatant was measured over time, as in (B). (F and G) Mouse primary WT and DogMo BMDM together with dog WT DH82 cells were primed with LPS (200 ng/mL for 3 h) and then stimulated with nigericin (20 μM for 1 h). The amounts of LDH and IL-1β were measured in the supernatant as in (B) and (C). (H and I) Mouse primary WT and DogMo BMDM were primed and stimulated as in (F) and (G). Total protein was precipitated from the lysate and gasdermin D cleavage was assessed by western blot analysis. Data are shown as means ± SEMs in (B) and (C) and (E)–(G). Data are pooled from 3 independent experiments in (B) and (C) and 2 independent experiments in (F) and (G), from 1 representative of 3 independent experiments in (D), from 1 representative of 2 independent experiments in (E), and from 1 single experiment in (H) and (I). Statistical significance was calculated by a 1-way ANOVA for each nigericin concentration individually followed by Tukey’s multiple comparison test in (B) and by 2-tailed unpaired t test for each nigericin concentration individually assuming equal or unequal variances in (C); ns, not significant; ^∗^p < 0.05, ^∗∗^p < 0.01, ^∗∗∗^p < 0.001. See also Figure S4.

An alternative explanation for our data is that species-specific differences in NLRs may account for the weak activation of canonical inflammasomes that we see in dog cells. AIM2, for example, is absent in many species, including Carnivora (Brunette et al., 2012). Analysis of the repertoire of NLR genes across the Carnivora identified that the bacterial sensors NLR family of apoptosis inhibitory proteins (NAIPs) and NLRC4 are predominantly missing or are pseudogenes in the Canidae, whereas the Felidae lack another bacterial sensor NLRP1 (Figure 3A) (Eckhart et al., 2009). The loss of these NLRs occurred early in the evolutionary trees of both the Canidae and Felidae (Figure S4A). This diversity in NLRs suggests that the lack of functional NAIP/NLRC4 in dogs would at least partially explain the altered inflammasome responses to *S*. Typhimurium we saw when comparing mouse and dog macrophages. Comparison of the cell death induced by *S*. Typhimurium in DH82 cells with mouse *Nlrc4*^*−/−*^ BMDM showed that both cell types resist rapid cell death (1 and 2 h), but by 6 h, *Nlrc4*^*−/−*^ BMDM are dying, whereas DH82 cells remain resistant (Figure 1C). NLRP3, a non-specific sensor of cellular insults (Swanson et al., 2019), in contrast, is conserved across all Carnivora. We next stimulated mouse WT, mouse *Casp1/11*^*−/−*^ iBMDM, and dog DH82 macrophages with increasing concentrations of the canonical NLRP3 activator nigericin. We saw that low concentrations of nigericin activated NLRP3-induced cell lysis in WT mouse iBMMs but not in DH82 cells (Figure 3B). This was not due to a slower induction of NLRP3-mediated inflammasome activation in DH82 cells, because when these cells were stimulated with this low concentration of nigericin over a period of 24 h, they remained viable, showing no cell death (Figure 3E). Cell lysis in dog cells could be induced by very high concentrations of nigericin, but this cell death was independent of both caspase-1 and -11 in mouse iBMDMs (Figure 3B). The failure of nigericin to cause inflammasome-induced cell lysis was not due to the inability of the caspase fusion protein to function per se because stimulation of the DogMo macrophages again showed cell lysis and gasdermin D cleavage, with minimal IL-1β production (Figures 3F–3I). Mouse WT BMDM produced IL-1β, as expected, in response to NLRP3-activating concentrations of nigericin, but dog cells produced no IL-1β until very high concentrations of nigericin were used (Figure 3C). Processed IL-1β was detected in the supernatant of DH82 cells stimulated with high concentrations of nigericin by western blot analysis, but only in relatively small amounts (Figure 3D).

**Figure 4:**
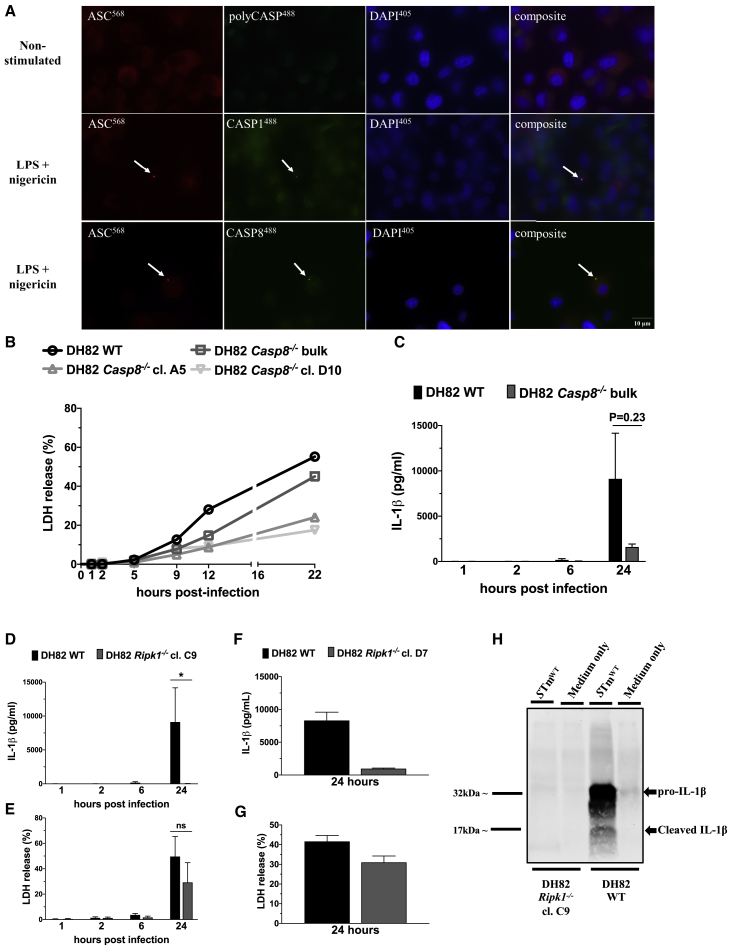
Caspase-8 mediates late cell death and IL-1β production in Carnivora cells infected with *S*. Typhimurium (A) Dog WT DH82 cells were primed with LPS (200 ng/mL) and stimulated with nigericin (20 μM for 1 h). Non-stimulated cells were left in cell culture medium. Live cells were stained for activated caspases (green, FLICA poly-caspase, FLICA caspase-1, or FLICA caspase-8). Following fixation, cells were stained for cytoplasmic ASC (red) and nuclei using DAPI staining (blue). White arrows point to ASC specks. (B) Dog WT, *Casp8*^*−/−*^ bulk edited, and cells from 2 individual *Casp8*^*−/−*^ DH82 clones were infected with *S*. Typhimurium MOI of 10, and the amount of LDH released in the supernatant was measured over time via a colorimetric assay. (C) Dog WT and *Casp8*^*−/−*^ bulk-edited DH82 cells were infected with *S*. Typhimurium MOI of 10, and the amount of IL-1β in the supernatant was measured over time by ELISA. (D and E) Dog WT and cells from 1 individual *Ripk1*^*−/−*^ DH82 clone were infected with *S*. Typhimurium MOI of 10, and the amounts of LDH and IL-1β were measured in the supernatant over time via colorimetric assay and ELISA, respectively. (F and G) Identical to (D) and (E), but with cells from a second individual *Ripk1*^*−/−*^ DH82 clone; LDH and IL-1β were measured in the supernatant at 24 h only. (H) Dog WT and cells from 1 individual *Ripk1*^*−/−*^ DH82 clone were infected with *S*. Typhimurium MOI of 1 and total protein extracts from cell lysates were subject to western blot analysis against dog-specific IL-1β. Uninfected controls (medium only) were also included for each cell line. Data are shown as means ± SEMs in (B)–(G). Data are pooled from 4 independent experiments in (C)–(E), from 1 representative of 3 independent experiments in (A), from 1 representative of 2 independent experiments in (B), and from 1 single experiment in (F)–(H). Statistical significance was calculated by the Mann-Whitney test in (C) and (D) and by 2-tailed unpaired t test assuming equal variances in (E); ns, not significant; ^∗^p < 0.05. See also Figure S4.

How is IL-1β processed in response to *S*. Typhimurium and nigericin in dog cells? Caspase-8 can be recruited to the inflammasome to process IL-1β and gasdermin D, particularly in the absence of caspase-1 (Lee et al., 2018; Man et al., 2013; Newton et al., 2019). We visualized ASC specks in DH82 cells stimulated with NLRP3-activating concentrations of nigericin and saw recruitment of both caspase-1 and caspase-8 fluorescent substrates, consistent with the presence of both caspase-1/-4 fusion protein and caspase 8 within the inflammasome complex (Figure 4A). We used CRISPR-Cas9 to delete the caspase-1/-4 gene from dog DH82 cells. Clones of DH82 cells lacking the caspase-1/-4 gene showed similar responses (cell death and IL-1β production) to WT DH82 cells after infection with *S*. Typhimurium (Figure S4B), or stimulation with NLRP3-activating concentrations of nigericin (Figure S4C). When, however, we used CRISPR-Cas9 to delete caspase-8 from dog DH82 macrophages, we saw impaired responses in *Caspase-8*^*−/−*^ bulk cells and individual *Caspase-8*^*−/−*^ clones infected with *S*. Typhimurium (Figures 4B and 4C). DogMo BMDM, but not their WT counterparts, also showed substantial resistance to early pyroptotic cell death induced by *Salmonella* when caspase-8 was pharmacologically inhibited (Figure S4D). These data collectively suggest that inflammasome formation in dog macrophages uses caspase-8, rather than the caspase-1/-4 fusion. Caspase-8 regulates the receptor-interacting serine/threonine-protein kinase 1 (RIPK1)/RIPK3 pathway that triggers necroptosis, but also regulates TLR4-dependent nuclear factor κ-light-chain enhancer of activated B cells (NF-κB)-driven transcription of genes such as pro-IL-1β. Carnivora cells cannot undergo necroptosis as the effector protein MLKL is missing from their genomes (Dondelinger et al., 2016). When we used CRISPR-Cas9 to inactivate RIPK1 from dog cells, IL-1β production was abolished in response to inflammasome stimulation (Figures 4D and 4F) without affecting cell death (Figures 4E and 4G). This RIPK1-dependent effect is most likely due to the loss of pro-IL-1β transcription driven by TLR4 priming from the *Salmonella* LPS (Figure 4H). This was confirmed by using the selective TLR4 inhibitor TAK242 on WT DH82 cells infected with *Salmonella*, which reduced IL-1β production without affecting cell death (Figure S4E). Collectively, our data suggest that what little inflammasome activation occurs in the Carnivora is predominantly driven by caspase-8, with any role for caspase-1/-4 being very minor.

### Inflammasome activity induces pore formation, but is uncoupled from cell lysis in the Carnivora

**Figure 5:**
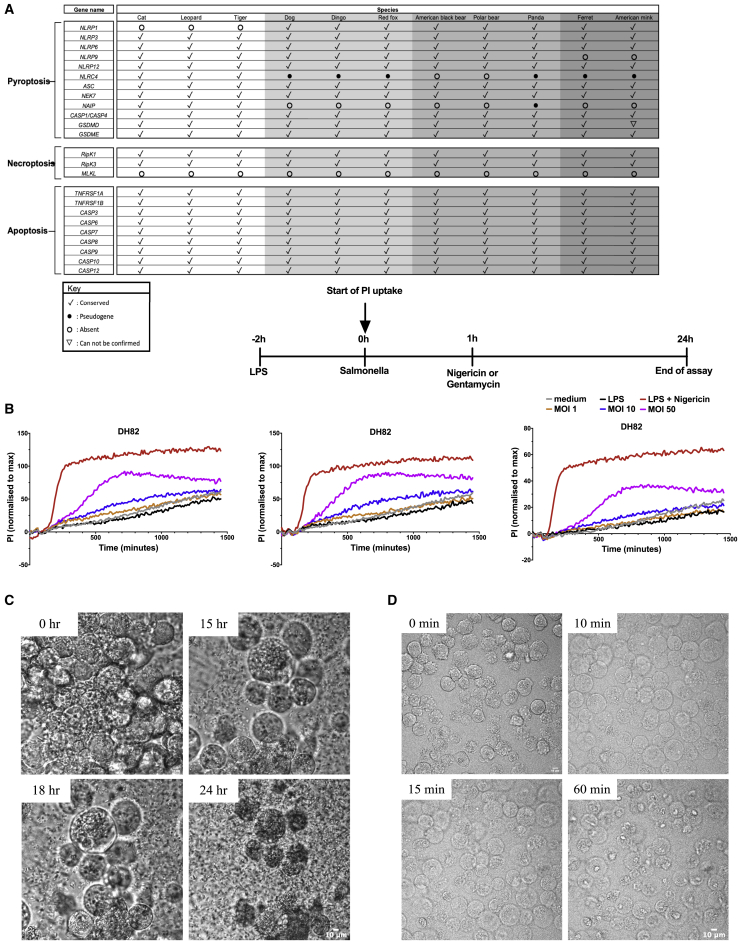
Inflammasome activity induces pore formation but is uncoupled from cell lysis in the Carnivora (A) Representative table showing the evolutionary conservation of main components of different lytic cell death pathways in species belonging to the Carnivora order. (B) Dog WT DH82 cells were infected with *S*. Typhimurium MOI of 1, 10, and 50 or primed with LPS (200 ng/mL) for 3 h and stimulated with nigericin (200 μM) for 24 h. Propidium iodide (PI) fluorescence was measured every 10 min over a 24-h period and expressed as %PI uptake normalized against maximum PI uptake achieved after lysing the cells with Triton X-100. Data from 3 independent experiments are shown. A simplified schematic of the experimental design is also shown. (C) Dog WT DH82 cells were infected with *S*. Typhimurium MOI of 50 for 24 h, and bright-field images were taken on a confocal microscope every 5 min over the 24-h period (see also Video S1). (D) Dog WT DH82 cells primed with LPS (200 ng/mL) for 3 h were stimulated with nigericin (200 μM) for 1 h and bright-field images were taken on a confocal microscope every minute over the 1-h period (see also Video S2). Representative images of the different morphological stages are shown. Data are shown as mean of 3 replicate wells for each independent experiment in (B). Data have originated from a single experiment in (C) and (D). Scale bar, 10 μm.

Our data suggest that in dogs and presumably in other Carnivora, non-canonical inflammasome activation is absent and canonical inflammasome activation is limited. In Carnivora there is a loss, or modification, of genes important in lytic cell death pathways (necroptosis and pyroptosis; Figure 5A). Gasdermin D pores can form, however, without inducing lytic cell death (Evavold et al., 2018), so as there are no antibodies available that cross-react with the dog gasdermin D protein, we measured propidium iodide (PI) uptake and used live cell imaging of DH82 cells to determine whether pyroptotic cell death pathways are completely missing in dog cells. *S.* Typhimurium-induced inflammasome activity classically processes gasdermin D to induce rapid lytic cell death in mouse or human cells, yet infected dog cells took up PI (Figure 5B), but appeared to be locked in the swollen phase and only ruptured very late during infection, presumably when the cell membrane could no longer contain the enormous intracellular bacterial load (Figure 5C; Video S1). In response to nigericin stimulation, DH82 cells again took up PI, suggesting that gasdermin D pores are formed (Figure 5B), but cells from these animals have a markedly reduced capacity for pro-inflammatory lytic cell death (Figure 5D; Video S2). The appearance of the caspase-1/-4 fusion and the loss of MLKL occurred early in the Carnivora evolutionary tree (Figure S4A). Gasdermin E, a protein that drives pyroptosis in response to caspase-3 activation (Wang et al., 2017), is conserved. The apoptotic caspases -3, -7, -8, and -9 are fully conserved across all Carnivora, suggesting that caspase-dependent cell death may be limited primarily to apoptosis pathways in these animals (Figure 5A). We do see some lytic cell death at very high doses of nigericin in dog cells (Figure 5D; Video S2), which could be driven by, for example, gasdermin E, but this occurs under conditions of limited physiological relevance.

Video S1. Dog DH82 cells are locked in the swollen phase and only rupture very late in infection with *S*. Typhimurium, related to Figure 5

Video S2. Dog DH82 cells have a markedly reduced capability for pro-inflammatory lytic cell death in response to nigericin stimulation, related to Figure 5

## Discussion

Here, we show that key inflammasome lytic cell death pathways thought to be critical for gut health in mammals are either genetically and/or functionally missing from the Carnivora. PI uptake without cell lysis occurs in dog cells in response to inflammasome activation, suggesting a dissociation of gasdermin D pore formation from lytic cell death. This is similar to the phenotype seen when the NINJ1 protein is deleted from human or mouse cells (Kayagaki et al., 2021), yet all Carnivora have NINJ1. Our data suggest that inefficient inflammasome activation occurs in dog cells such that insufficient gasdermin D pores form in the cell membrane to drive cell lysis, although the pores that are formed, as indicated by the PI uptake, should facilitate cytokine release (Evavold et al., 2018). Inflammasome-driven lytic cell death is, therefore, lost in Canidae, and this is particularly interesting because the Carnivora also lack the necroptotic effector MLKL, such that two of the critical inflammatory cell death pathways that are thought to be essential for host protection against infection are absent (Table 1). One of the key functions for inflammatory cell death is to protect the gut against infection (Crowley et al., 2020; Rauch et al., 2017; Schwarzer et al., 2020; Sellin et al., 2014; Tummers et al., 2020), yet canonical inflammasome, non-canonical inflammasome, and necroptotic lytic cell death pathways in Carnivora are inactive. All Carnivora, even the giant panda, consume a high-protein diet (Nie et al., 2019). Emerging ecological evidence suggests that a high-protein diet has antimicrobial properties (Cotter et al., 2019), and we speculate that the evolutionary and functional reduction in inflammasome activation in the Carnivora may simply reflect the protection against infection conferred to these animals by their dietary habits. This hypothesis is supported by different lines of evidence. Both dogs and cats can asymptomatically carry *Salmonella* (Gow et al., 2009; Leonard et al., 2011). Diarrhea in dogs, often associated with bacterial enteropathogens, is one of the most common clinical conditions encountered by veterinarians (Marks and Kather, 2003), and *Shigella* infection of mice lacking NAIP/NLRC4 in their intestinal epithelial cells induces a bacillary dysentery (Mitchell et al., 2020).

**Table 1:** Summary of cell death pathways, with those missing in Carnivora shown in parentheses

	Pyroptosis	Necroptosis	Apoptosis
Sensor	Many, e.g., NLRP3 (NLRP1, NLRC4, AIM2)	many, e.g., TLR4	many, e.g., BCL protein family
Adaptor	ASC	many, e.g., TRIF, RIPK1	APAF-1
Processor	(caspase-1), (caspase-11/-4), caspase-1/-4 fusion	RIPK3	caspase-8, caspase-9, (caspase-1)
Effector	gasdermin D	(MLKL)	caspase-3, caspase-7

Canonical inflammasomes traditionally couple a receptor, such as an NLR or AIM2, with ASC and the effector protein caspase-1, whereas non-canonical inflammasomes are driven by caspase -4/-5/-11 to indirectly activate NLRP3 (Broz and Dixit, 2016). In the Carnivora, caspase-1 and -4 are fused into a single caspase that has the CARD of caspase-1 but the catalytic site of caspase-4/-11. This arrangement should result in a protein that is unable to bind to LPS and process pro-IL-1β, but can efficiently process gasdermin D. Macrophages from a mouse expressing an analogous caspase-1/-11 fusion fulfilled these predictions, although when expressed as a recombinant protein, the dog caspase-1/-4 can process both gasdermin D and pro-IL-1β *in vitro*. In the dog macrophages, however, the caspase-1/-4 fusion is relatively inactive, producing no lytic cell death and limited gasdermin D pore formation, and what little pro-IL-1β processing and cell death occurs is through caspase-8. This caspase can be recruited to canonical inflammasomes, particularly in the absence of caspase-1, to process pro-IL-1β and drive cell death (Man et al., 2013; Newton et al., 2019), with our data fully supporting this role for caspase-8 in the Carnivora.

IL-1β is active in domestic members of the Carnivora, such as dogs (Dinarello, 2018), and the genes for IL-1β are well conserved in these animals compared to other mammals (Soller et al., 2007). The importance of this cytokine in the inflammatory responses of these mammals is less clear, although it is assumed to be essential. The low level of inflammasome-mediated caspase-1 activity may not compromise pro-IL-1β cleavage because other enzymes can cleave this cytokine, for example, caspase-8 (as shown here), neutrophil elastase, proteinase-3, cathepsin G, and chymase (Afonina et al., 2015), all of which should be active in the Carnivora. Pro-IL1β release, for example, through the pores formed by gasdermin D, under inflammatory conditions into the extracellular environment where many different cell types are present, should result in neutrophil- and mast cell-derived protease-dependent activation of IL-1β (Afonina et al., 2015). Our data (Figure 5) suggest that gasdermin D pores form because PI is taken up into the cells, so this will, presumably, facilitate the release of pro-IL-1β and/or IL-1β cleaved by caspase-8. Other members of the IL-1 family could also help compensate for low levels of active IL-1β. IL-1α, for example, is well conserved between mammals, has a similar range of biological activities, and can be cleaved by calpain, neutrophil elastase, granzyme B, and chymase (Afonina et al., 2015).

There are potential consequences for the loss of the inflammasome and cell death pathways, particularly in organ systems other than the gut. The presence of bacteria in other organs of the Carnivora may be expected to lead to either immune deficits and/or allow the pathogen to hide. Inflammasome-induced pyroptosis and subsequent lytic cell death are thought to be critically important mechanisms by which pathogens are controlled in the host (Martinon et al., 2009; Miao et al., 2010). The lack of pyroptotic cell lysis in the Carnivora should, therefore, severely compromise the susceptibility of these animals to infection, yet these mammals continue to thrive. The lack of functional NAIP/NLRC4 in the Canidae should affect the recognition of a number of bacterial pathogens, including *Salmonella*, *Pseudomonas*, and *Bacillus*. The fact that Felidae lack NLRP1, which is activated by protease cleavage and thus is susceptible to processing by bacterial or viral proteases (Chui et al., 2019; Sandstrom et al., 2019), potentially compromises the immune status of these animals in response to pathogens. Our data suggest that the Carnivora also have a limited capacity to activate the NLRP3-dependent inflammasome pathway. The compromise in inflammasome functionality in the Carnivora does not seem to predispose them to infection; however, another possible consequence could be to allow pathogens to reside undetected and therefore facilitate the potential for zoonotic carriage. One reason for the high zoonotic carriage by bats has been suggested to be due to the dampened activation of NLRP3 (Ahn et al., 2019). The muted inflammasome activation we see in Canidae may also facilitate zoonotic carriage. COVID-19, for example, is carried by mink (Oude Munnink et al., 2021), and although the genome of this species is incomplete, the caspase-1 locus is intact with, as expected, a predicted caspase-1/-4 fusion protein consistent with poor inflammasome functionality. The loss of these immune pathways in Carnivora may, therefore, have the unexpected consequence of permitting certain pathogens to evade host detection when in habitats other than the gut, thus facilitating the carriage of zoonotic infections.

## STAR★Methods

### Key resources table

**Table:** 

REAGENT or RESOURCE	SOURCE	IDENTIFIER
**Antibodies**		

Rabbit polyclonal anti-human, mouse ASC (AL177)	AdipoGen	Cat#AG-25B-0006-C100; RRID: AB_2885200
Goat polyclonal anti-canine IL-1 beta / IL-1f2	R&D Systems	Cat#AF3747, RRID: AB_2124605
Rabbit polyclonal anti-mouse, rat caspase-1 p10 (M-20)	Santa Cruz Biotechnology	Cat# sc-514, RRID: AB_2068895
Rat monoclonal anti-mouse, rat caspase-11 (17D9)	Santa Cruz Biotechnology	Cat# sc-56038, RRID:AB_781818
goat polyclonal anti-rabbit IgG-HRP	Santa Cruz Biotechnology	Cat#sc-2054, RRID:AB_631748
Goat polyclonal anti-rat Rat IgG-HRP	Novus	Cat#NB-7115, RRID:AB_524662
mouse monoclonal anti-rabbit IgG-HRP	Santa Cruz Biotechnology	Cat#sc-2357, RRID:AB_628497
Mouse monoclonal IgGκ BP-HRP	Santa Cruz	Biotechnology Cat# sc-516102, RRID:AB_2687626
Goat anti-rabbit IgG Alexa Fluor 488	ThermoFisher Scientific	Cat#A-11034, RRID:AB_2576217
Goat anti-rabbit IgG Alexa Fluor 568	ThermoFisher Scientific	Cat#A-11036, RRID:AB_10563566

**Bacterial and virus strains**		

*Salmonella* Typhimurium strain SL1344	This paper	N/A

**Biological samples**		

Canine PBMC, for donor details see Table S1	This paper	N/A

**Chemicals, peptides, and recombinant proteins**		

Hoechst 33342	ThermoFisher Scientific	Cat#H3570
Fetal Bovine Serum	ThermoFisher Scientific	Cat#16000044
TAK-242 TLR4 Inhibitor	Sigma-Aldrich	Cat#5083360001
Ultrapure LPS, *E. coli* 0111:B4	Invivogen	Cat#tlrl-3pelps
Pam3CSK4	Invivogen	Cat#tlrl-pms
Nigericin sodium salt	Sigma-Aldrich	Cat#N7143
FUGENE HD Transfection Reagent	Promega	Cat#E2311
Caspase-8 Inhibitor Z-IETD-FMK	R&D Systems	Cat#FMK007
Cas9 endonuclease	IDT	Cat#1074182
Protease Inhibitor Cocktail	Sigma-Aldrich	Cat#P8340
Propidium Iodide-1 mg/mL Solution in Water	ThermoFisher Scientific	Cat#P3566
Z-Val-Ala-Asp-FMK	Enzo Life Sciences	Cat# ALX-260-020
Ac-Trp-Glu-His-Asp-AFC trifluoroacetate salt	Bachem	Cat# 4089441
Ac-Trp-Thr(Bzl)-His-Asp-ACC	Ramirez et al., 2018	N/A
Ac-Tle-Bpa-His(Bzl)-Asp-ACC	Ramirez et al., 2018	N/A

**Critical commercial assays**		

FAM FLICA Caspase-1	Bio-Rad	Cat#ICT097
FAM FLICA Caspase-8	Bio-Rad	Cat#ICT099
FAM FLICA Poly Caspase	Bio-Rad	Cat#ICT091
Mouse 1L-1β BD OptEIA ELISA	BD Biosciences	Cat#559603
Canine 1L-1β/IL-1F2 Duoset	R&D Systems	Cat#DY3747
MiSeq Reagent Kit v2	Illumina	Cat#MS-103-1001
QuantiTect Reverse Transcription kit	QIAGEN	Cat#205311
PowerUp SYBR Green Master Mix	ThermoFisher Scientific	Cat#A25741
Pierce BCA Protein Assay	ThermoFisher Scientific	Cat#23227
Cytotox 96 Non-Radioactive Cytotoxicity Assay	Promega	Cat#G1780

**Deposited data**		

All source data associated with paper	This paper	https://doi.org/10.17632/pph3mdbrw7.1

**Experimental models: Cell lines**		

Murine iBMDM (wild-type, *Casp1/11*^*−/−*^ and *Nlrc4*^*−/−*^*)*	Fitzgerald Lab, UMASS Medical School	N/A
Canine DH82 wild-type	ATCC	Cat#CRL-10389
Canine DH82 *Casp1/4*^*−/−*^, *Casp8*^*−/−*^, *Ripk1*^*−/−*^	This paper	N/A

**Experimental models: Organisms/strains**		

Mouse C57BL/6N: Caspase 1/4 DogMo allele (*Casp1/4* in-frame fusion, 20.5 kb genomic deletion)	This paper	N/A
Mouse CB7BL/6NCrl	Charles River Laboratories	632C57BL/6J

**Oligonucleotides**		

sgRNA target sequence in mouse *Casp1* intron 4: 5′-AATTTAGATCAACACTAGGA-3′	This paper	N/A
sgRNA target sequence in mouse *Casp4* intron 3: 5′-GGAACTTTGACTAGGTACTA-3′	This paper	N/A
Primers for real-time quantitative PCR, see Table S2	This paper	N/A
sgRNA target sequence in dog *Casp8* exon 7: 5′-TTTTATTCAGGCTTGTCAAG-3′	This paper	N/A
sgRNA target sequence in dog *Casp8* exon 7: 5′-CCTACCGAAACCCCAATGGAG-3′	This paper	N/A
sgRNA target sequence in dog *Ripk1* exon 3: 5′-TAATTATGGAGACCATTGAA-3′	This paper	N/A
sgRNA target sequence in dog *Ripk1* exon 3: 5′-TGGAGAAGGCGTAATACACA-3′	This paper	N/A
sgRNA target sequence in dog *Casp1/4/11* exon 6: 5′-AACCTCAAGGACAAACCGA-3′	This paper	N/A
sgRNA target sequence in dog *Casp1/4/11* exon 6: 5′-GCATCCTGAATGGAATCTGT-3′	This paper	N/A

**Recombinant DNA**		

pET29b(+)-dog caspase-1/-4 delta CARD C-terminal 6 × His tag	This paper	N/A
pET29b(+)-mouse caspase-1 delta CARD C-terminal 6 × His tag	Ramirez et al., 2018	N/A
pET29b(+)-mouse caspase-11 delta CARD C-terminal 6 × His tag	Ramirez et al., 2018	N/A
pET29b(+)-mouse pro-IL1β C-terminal 6 × His tag	Ramirez et al., 2018	N/A
pET15b-mouse gasdermin D N-terminal 8 × His tag	Bibo-Verdugo et al., 2020	N/A

**Software and algorithms**		

MARS data analysis software	BMG Labtech	https://www.bmglabtech.com/it/mars-data-analysis-software/
Prism 7	GraphPad	https://www.graphpad.com/scientific-software/prism/
Prism 8	GraphPad	https://www.graphpad.com/scientific-software/prism/
Ensembl search	Ensembl genome browser	http://www.ensembl.org//useast.ensembl.org/index.html?redirectsrc=//www.ensembl.org%2Findex.html
Ensembl BLASTn	Ensembl genome browser	http://www.ensembl.org//useast.ensembl.org/Multi/Tools/Blast?redirectsrc=//www.ensembl.org%2FMulti%2FTools%2FBlast
BioEdit v7.2	BioEdit	https://bioedit.software.informer.com/7.2/
RStudio v1.2.5001	RStudio	https://www.rstudio.com/
Figtree v1.4.4	Figtree	http://tree.bio.ed.ac.uk/software/figtree/
CRISPR Guide RNA Design tool	Benchling	https://www.benchling.com/
Alt-R CRISPR-Cas9 Guide RNA tool	IDT	https://www.idtdna.com/site/order/designtool/index/CRISPR_CUSTOM?c=US
Outknocker online analysis tool	Outknocker	http://www.outknocker.org/outknocker2.htm

### Resource availability

#### Lead contact

Further information and requests for resources and reagents should be directed to and will be fulfilled by the lead contact, Clare E. Bryant (ceb27@cam.ac.uk).

#### Materials availability

All unique/stable reagents generated in this study are available from the lead contact with a completed materials transfer agreement.

### Experimental model and subject details

#### Cell lines

All cells (cell lines and primary) used for this work were maintained at 37°C, 5% CO_2_. Murine immortalized bone marrow derived macrophages (iBMDMs), a gift from the laboratory of Kate Fitzgerald (UMASS Medical School, USA), and the canine malignant histiocytic macrophage-like cell line DH82 (ATCC) were cultured in complete DMEM (Sigma) containing 10% FBS (ThermoFisher), 5 mM L-glutamine (Sigma) and supplemented with 100 μg/ml streptomycin and 100 units/ml of penicillin (Sigma).

#### Primary cell cultures

Primary mouse BMDMs were prepared from male and female 8-24 week-old wild-type C57BL/6N (Charles River) and “Dog-Mouse” (DogMo) chimeric mice, a gift from the laboratory of Vishva Dixit (Genentech, USA). Wild-type mice were maintained in a specific pathogen-free facility under the UK Home Office Project License number P48B8DA35 and complied with the University of Cambridge Ethics Committee regulations. All breeding and maintenance of the DogMo chimeric mice were conducted under protocols approved by the Genentech Institutional Animal Care and Use Committee in an Association for Assessment and Accreditation of Laboratory Animal Care (AAALAC)-accredited facility in accordance with the Guide for the Care and Use of Laboratory Animals and applicable laws and regulations.

Bone marrow cells were isolated from tibias and femurs and cultured in DMEM containing 10% FCS, 8 mM L-glutamine, 20% L929 conditioned medium (complete primary DMEM) supplemented with 100 μg/ml streptomycin and 100 units/ml penicillin for 6 days before use. Fresh complete primary DMEM supplemented with antibiotics was added at day 3 post-isolation.

Peripheral blood mononuclear cells (PBMCs) were taken from residual blood samples from dogs admitted to the Queen’s Veterinary School Hospital (Department of Veterinary Medicine, University of Cambridge) and pooled (donor details are given in Table S1). Mononuclear cells (MNCs) were isolated using Histopaque-1077 density gradient medium (Sigma) and SepMate mononuclear cell tubes (Stem Cell Technologies).

#### Microbial strains

Frozen glycerol stocks of *Salmonella* Typhimurium (*S*. Typhimurium) were streaked onto LB agar (Sigma) and incubated for 24 hours at 37°C. Single colonies were then selected and inoculated fresh LB agar broth (Sigma). Liquid cultures were incubated under shaking (200 rpm) for 17.5-18 hours at 37°C. They were then diluted 1:10 in fresh LB agar broth and incubated for an additional 2 hours to reach logarithmic growth before applied onto cells.

### Method details

#### Carnivora gene presence/absence analysis

Genomes of the Carnivora species listed on the Ensembl genome database were examined for the presence of key innate immune system and cell death genes. Genes were first assessed for presence using the standard Ensembl annotation tool. For genes that were absent, we extracted the closest evolutionary relative’s cDNA splice variants (or the human cDNA variants if no relatives could be found in Carnivora) and performed Blastn **(**http://www.ensembl.org//useast.ensembl.org/Multi/Tools/Blast?redirectsrc=//www.ensembl.org%2FMulti%2FTools%2FBlast**)** on release 98 of the Ensembl genome database. In some cases, the gene was found but not appropriately annotated. Standard checks were performed to confirm that the gene was a true ortholog of the gene in question. These included 1) producing a bootstrapped neighbor-joining phylogeny and confirming that the gene tree was consistent with the species phylogeny and 2) checking that the gene length was consistent with not containing premature stop codons. The phylogenetic tree was produced in RStudio (v1.2.5001) using the ‘ape’ and ‘seqinr’ packages. The function ‘dist.dna(x, model = ”F84”)’ first produced a matrix of pairwise distances from the DNA, ‘nj()’ constructed a tree from this distance matrix and bootstrapping of the tree was carried out using the function ‘boot.phylo()’. A bootstrap value of > 80% was taken as support for a particular node. Genes may be missing from genomes due to errors in genome assembly. To verify that genes not found by annotation and Blastn were due to true absence, synteny between the genomes was examined. A queried species genome and the most closely related species genome available containing the investigated gene were compared. The gene coordinates were retrieved from the “Region in detail” tool on Ensembl’s gene location option, this allowed the gene size, location and relation to nearby genes to be obtained. Using this information, synteny maps were generated for species in which the gene is thought to be deleted.

#### Infection with *S.* Typhimurium

Cells were seeded in 96-well flat-bottom plates at a concentration of 1x10^6^ cells/ml in complete DMEM and incubated overnight at 37°C in 5% CO_2_. *S*. Typhimurium SL1344 was grown for 18h before sub-culturing to logarithmic growth for 2h. Cells were infected with *S*. Typhimurium SL1344 for 1 hour with multiplicity of infection (MOI) 1, 10 or 50. For long time courses (over 2 hours) cell culture medium was replaced with complete DMEM supplemented with gentamicin (Fisher Scientific) at 50 μg/ml final concentration and further incubated for one hour at 37°C in 5% CO_2_. For the 6- and 24-hour time-points, medium containing 50 μg/ml gentamicin was replaced with fresh complete DMEM containing gentamicin at a final concentration of 10 μg/ml. At each time point the supernatant was removed and stored at −80°C until required. In time lapse experiments medium was not replaced to ensure that the field of vision was retained. For blocking TLR4 activation, TAK 242 (Sigma) was added to the cells 1 hour before infection with *Salmonella* at a final concentration of 1 μM and re-supplemented every time the cell culture medium was changed.

#### Inflammasome activators and inhibitors

Cells were seeded at 1x10^6^ cells/ml and incubated overnight at 37°C, 5% CO_2_. Adherent cells were primed with either ultrapure *E.coli* lipopolysaccharide (LPS) (InvivoGen) at a final concentration of 200 ng/ml for 3 hours or Pam3CSK4 (Pam3) (InvivoGen) at a final concentration of 10 μg/ml for 4 hours at 37°C, 5% CO_2_. Cells were stimulated with nigericin (Sigma-Aldrich) at a final concentration of 20 μM to 200 μM for 1 hour or at a final concentration of 10 μM for 24 hours. For non-canonical inflammasome activation, cells were incubated with ultrapure LPS at 5 μg/ml to 20 μg/ml final concentration in the presence of FuGENE HD transfection reagent (Promega) for 16 hours at 37°C, 5% CO_2_. LPS and transfection reagent were pre-incubated for 15 minutes at room temperature prior to addition to the cells. For pharmacological inhibition of caspase-8, cells were pre-incubated with the caspase-8 Z-IETD-FMK inhibitor (R&D Systems) at a final concentration of 10 μM and and re-supplemented every time the cell culture medium was changed.

#### Genome editing

The crRNA designs were performed using Benchling online research platform (https://benchling.com/faq) and crRNA sequences were confirmed using IDT’s Custom Alt-R® CRISPR-Cas9 guide RNA tool (https://www.idtdna.com/site/order/designtool/index/CRISPR_CUSTOM?c=US). Equimolar amounts of tracr- and crRNAs (IDT) were mixed and heated at 95°C for 5 minutes. Ribonucleoprotein (RNP) assembly reaction was performed on duplexes by adding an equimolar concentration of Cas9 endonuclease protein (IDT) to the complex. The RNP complex was then supplemented with equimolar concentration of Alt-R® Cas9 electroporation enhancer (IDT). DH82 cells were resuspended in Nucleofector Solution V containing electroporation supplement (Lonza) and 10 μL of RNP was introduced. The nucleofection buffer-cell-RNP mix was then transferred directly into an electroporation cuvette and a single electroporation was performed in an Amaxa Nucleofector 2b (Lonza) using the X-005 program. Cells were rested for 48 hours, after which they were seeded into 96 well plates at single-cell dilution to obtain single cell clones. Monoclones were genotyped as previously described (Gram et al., 2021). Briefly, locus-specific primers were designed based on the canine reference genome (CanFam3.1) using Primer-blast and were designed to incorporate 5′ adaptor sequences. First level PCR was performed using genomic DNA extracted from monoclones and the locus-specific primers. Second level MiSeq PCRs were performed using the first round PCR product as template and the universal 96-well MiSeq barcode primers, prepared by combining 8 unique forward and 12 unique reverse primers. Second level MiSeq PCR products were pooled, cleaned up and subjected to sequencing using the Illumina MiSeq Reagent Nano Kit v2 300-cycles, #MS-103-1001) according to manufacturer’s instructions on the MiSeq benchtop sequencing systems (Illumina). Loss of gene expression was confirmed by qPCR analysis (Figure S5).

#### Analysis of sequencing output fastq files

Sequences were analyzed using the OutKnocker online analysis tool (http://www.outknocker.org/outknocker2.htm).

#### Real-time quantitative PCR

Total RNA was extracted from cell pellets using the RNeasy Plus Mini Kit together with QIAshredder columns (QIAGEN). Isolated RNA was subjected to genomic DNA removal and reverse transcription into cDNA using the QuantiTect Reverse Transcription Kit (QIAGEN). Real-time quantitative PCR was performed on an Applied Biosystems 7500 Fast Real-Time PCR System using PowerUp SYBR Green Master Mix (Thermo Fisher Scientific) and gene-specific primers (Table S2). Relative gene expression was determined using the comparative CT method after normalization to two reference genes (*GAPDH* and *HPRT1*) and expressed relative to WT DH82s.

#### Total protein extraction of cell lysates and cell culture supernatants

Cell pellets were disrupted in ice-cold RIPA solution (150 mM NaCl, 10 mM Tris-HCl, 5 mM EDTA, 1% Triton X-100, 10 mM NaF, 1 mM NaVO_4_, 20 mM PMSF) supplemented with Protease Inhibitor Cocktail (Sigma-Aldrich) at 1:100 dilution and incubated for 30 minutes on ice. Lysed cells were centrifuged at 14,000 rpm for 15 minutes at 4°C. Protein concentration was determined using the Pierce BCA Protein Assay Kit (Thermo Fisher). Standardized amounts of each cell lysate were prepared for immunoblotting by incubating the appropriate volume of sample with Pierce Lane Marker (5x) Reducing Sample Buffer (Thermo Fisher) for 10 minutes at 100°C. After denaturation, lysates were cooled on ice followed by brief centrifugation and subjected to SDS-PAGE.

#### Total protein extraction from cell culture supernatants

Total protein was extracted from clarified supernatants using methanol/chloroform extraction method. Samples were then centrifuged at 16,000 x g for 12 minutes at 4°C. The intermediate phase containing the precipitated protein was carefully aspirated and centrifuged for 10 minutes at 4°C. Protein pellets were washed twice in ice-cold methanol. In between washes the resuspended pellets were centrifuged at 16,000 x g for 5 minutes at 4°C. After the final wash, the methanol was carefully removed, and the pellets were allowed to dry. Pellets were re-suspended in Laemmli 2 x concentrate sample buffer (Sigma Aldrich), heated at 100°C for 10 minutes and stored at −80°C until required.

#### Western blotting

Denatured proteins were separated by SDS-PAGE using 12% tris-glycine polyacrylamide gels and subsequently transferred to nitrocellulose membranes followed by incubation with primary (anti-ASC, anti-canine IL-1β, anti-caspase-1 p10, anti-caspase-11 or anti-GSDMD) and HRP-conjugated secondary antibodies. Proteins were detected using Western Lightning Plus-ECL Substrate (Perkin Elmer) and protein bands were visualized using a GeneGnome chemiluminescence imager (Syngene, GeneGnome XRQ).

#### LDH release quantification

Supernatants were assayed for LDH release at indicated time points using the CytoTox 96 Non-Radioactive Cytotoxicity Assay kit (Promega) per the manufacturer’s instruction. Absorbance values (absorbance 490 nm and 680 nm) were measured on either a PHERAstar or CLARIOstar Microplate Reader (both BMG Labtech). Percentage cell death values are expressed relative to total LDH release induced by detergent lysis.

#### Propidium iodide uptake

DH82 were seeded into 96-well black/clear bottom microplates at 5 × 10^4^ cells/well in complete DMEM and incubated overnight. Cells were either unprimed or primed with 200 ng/ml LPS for 3 hours prior to stimulation with 100 μM nigericin. Cells were infected with *Salmonella* at the indicated MOIs in imaging medium (Opti-MEM containing GlutaMax, HEPES and 10% FBS). Propidium iodide (PI) was used at a final concentration of 2 μg/ml. Triton X-100 (0.2% w/v in imaging medium) was added to control wells. Measurements were taken every 10 minutes on a CLARIOstar microplate reader set to 37°C. After the first kinetic window (1 hour), the plate was removed and 100 μM nigericin added to the LPS-primed wells, or in the *Salmonella*-infected wells, the medium was replaced with imaging medium supplemented with 50 μg/ml gentamicin, and fluorescence readings recorded every 10 minutes for a further 23 hours. The percentage PI uptake was normalized against the unstimulated fluorescence recordings at the start of each kinetic window (0%) and the maximal PI uptake (100%) induced by Triton X-100.

#### Cytokine measurement using ELISA

Mouse IL-1β and canine IL-1β released into cell culture supernatant was measured using Mouse IL-1β ELIOptEIA (BD Biosciences) and Canine IL-1β/IL-1F2 DuoSet (R&D Systems) respectively, as described in the manufacturer’s instructions. Absorbance was read at 450 nm wavelength (570 nm correction) using either a PHERAstar or CLARIOstar Microplate Reader (BMG Labtech).

#### Sample preparation for immunofluorescent staining and imaging

Murine and canine iBMDMs and primary macrophages were seeded at a density of 2x10^5^ cells/well on an 8-well chamber slide (Thermo Fisher) and incubated overnight at 37°C, 5% CO_2_. Following ligand stimulation, cells were incubated with either FAM FLICA caspase-1, caspase-8 or poly-caspase (all Bio-Rad) according to the manufacturer’s instructions. Post stimulation, cells were fixed in 4% paraformaldehyde in PBS (Thermo Fisher Scientific) and incubated with anti-ASC (AL177) pAb (Adipogen) followed by incubation with goat-anti-rabbit-IgG-AlexaFluor488 (Invitrogen) or -AlexaFluor568 (Invitrogen) secondary antibodies. Cells were counterstained with nuclear Hoechst 33342 (Invitrogen) labeling solution followed by mounting in VECTASHIELD Antifade Mounting Medium (Vector) and imaged using an Inverted Fluorescence Microscope (Leica DM IRM).

#### Recombinant protein expression and *in vitro* caspase activity assays

Constructs encoding mouse pro-IL-1β, as well as CARD deleted modified versions of caspase-1 and caspase-11 in pET29b(+) containing a C-terminal 6 × His tag were previously described (Ramirez et al., 2018). The DNA encoding the CARD deleted version of dog caspase-1/-4 containing a C-terminal 6 × His tag was purchased from Integrated DNA technologies (IDT, San Diego, CA) and cloned into pET29b(+) by using Nde I and Xho I. Gasdermin D sequence was amplified from a pET29b(+) construct using primers to add an N-term 8xHis and cloned into pET15b using Nde I and Xho I restriction enzymes (Bibo-Verdugo et al., 2020). Proteins were expressed in BL21(DE3) *E. coli* cultures and purified as previously described (Ramirez et al., 2018). Protein concentration of pro-IL-1β and gasdermin D was calculated by absorbance at 280 nm and the concentration of caspases was calculated by active site titration with z-VAD-fmk. Activity assays were performed in 100 μL final volume and assay buffer consisted of 20 mM PIPES, 10% sucrose, 100 mM NaCl, 0.1% CHAPS, 1 mM EDTA, 10 mM DTT and 0.75 M sodium citrate. The fluorogenic peptide substrates Ac-Trp-Thr(Bzl)-His-Asp-ACC and Ac-Tle-Bpa-His(Bzl)-Asp-ACC and Ac-WEHD-AFC was purchased from BACHEM. For *k*cat/KM determination, the concentration of fluorogenic peptide substrates varied in the range of 5-200 μM. Reactions were monitored for 30 minutes at 37°C in a CLARIOstar plate reader (BMG LabTech). The ACC fluorophore was detected at excitation/emission 355/460 nm and AFC at 400/505 nm. Reaction velocity was calculated using MARS data analysis software (BMG LabTech) and the kinetic parameters with Prism 7 (GraphPad) using the Michaelis-Menten equation. Recombinant inflammatory caspases were subjected to 2-fold dilution series and incubated for 30 minutes at 37°C with 4 μM gasdermin D or pro-IL1β. Reactions in a 60 μL final volume were performed using assay buffer minus sodium citrate. After incubation, reactions were terminated by the addition of 30 μL of 3 × SDS loading buffer and heated at 95°C for 5 minutes. Reaction products were separated on 4%–12% Bis-Tris polyacrylamide gels and stained with Instant Blue (Expedeon).

#### Generation of a mouse with a Casp1-Casp4 gene fusion

To generate a mouse allele corresponding to the dog caspase gene fusion, a CRISPR strategy with two sgRNAs was used to generate a 20,524 bp deletion (GRCm38/mm10 chr9:5,302,869-5,323,392). The 5′ sgRNA (binding to chr9:5,302,865-5,302,884 reverse strand) is located in *Casp1* intron 4 and the 3′ sgRNA (binding to chr9:5,323,374-5,323,393) is located in *Casp4* intron 3 (Figure S2A). The deletion thus created an in-frame fusion between *Casp1* exons 1-4 and *Casp4* exons 4-9, resulting in a fusion protein similar to the dog fusion protein (Figure S2B). Cas9 mRNA and synthetic sgRNAs (Synthego) were co-microinjected into C57BL/6N mouse zygotes and resulting mosaic founders positive for the large deletion were analyzed for absence of off-targets essentially as described and subsequently bred to C57BL/6N for transmission of the fusion allele. Homozygous mice were used in this study (Anderson et al., 2018; Sievers et al., 2011).

### Quantification and statistical analysis

Unless stated otherwise, all standard statistical analyses were performed using Prism software v.7 or v.8 (GraphPad). Statistical details such as definition of the center and dispersion of the data, exact value of n and what n represents and statistical tests used are described in the appropriate figure legend. Statistical significance was defined as ns = not significant, ^∗^ = p < 0.05, ^∗∗^ = p < 0.01 and ^∗∗∗^ = p < 0.001.

## Data Availability

All source data used to generate the main and supplementary figures presented in this paper have been deposited at Mendeley and are publicly available as of the date of publication with the Mendeley Data: https://doi.org/10.17632/pph3mdbrw7.1 This paper does not report original code. Any additional information required to reanalyse the data reported in this paper is available from the lead contact upon request.

## Supplemental information

Document S1. Figures S1–S5 and Tables S1 and S2

Document S2. Article plus supplemental information
