# Supplementary material for: Evolutionary loss of inflammasomes in the Carnivora and implications for the carriage of zoonotic infections
Source: Cell Rep. 2021 Aug 24;36(8):109614. doi: 10.1016/j.celrep.2021.109614 (PMC8411117; doi:10.1016/j.celrep.2021.109614)
Supplement: Document S1. Figures S1–S5 and Tables S1 and S2 [file mmc1.pdf]

Cell Reports, Volume *36*

Supplemental information

Evolutionary loss of inflammasomes in the Carnivora and implications for the carriage of zoonotic infections

Zsofi Digby, Panagiotis Tourlomousis, James Rooney, Joseph P. Boyle, Betsaida Bibo-Verdugo, Robert J. Pickering, Steven J. Webster, Thomas P. Monie, Lee J. Hopkins, Nobuhiko Kayagaki, Guy S. Salvesen, Soren Warming, Lucy Weinert, and Clare E. Bryant

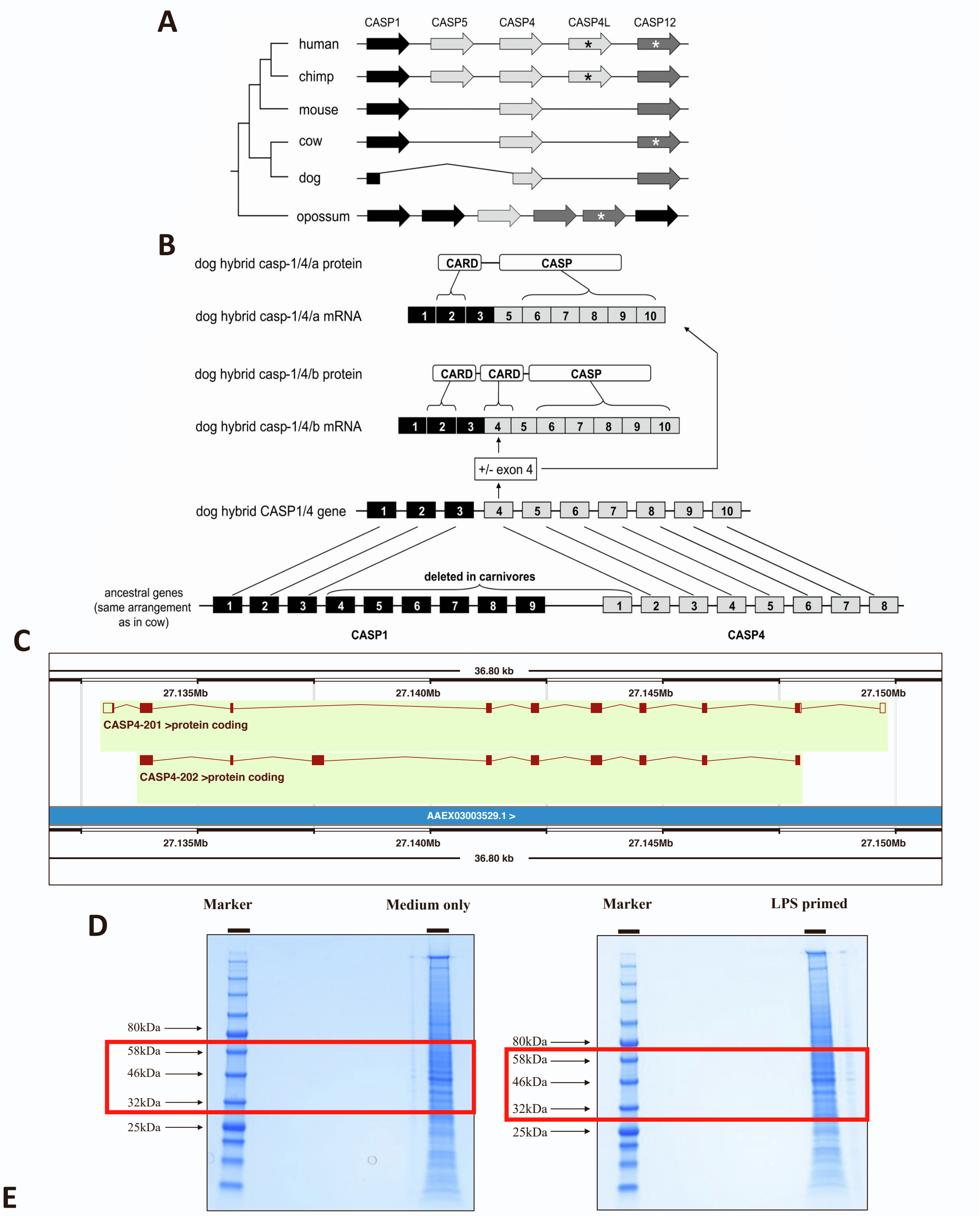

E

Cell type	Stimulation	Protein hit	Mass	Score	Matches	Sequences	emPAI	OS	OX	GN	PE	SV
DH82	Medium only	F1PBR9_CANLF	46294	50	3(2)	2(1)	0.11	Canis lupus familiaris	961 5	CASP4	3	2
DH82	LPS primed (200 ng/ml)	F1PBR9_CANLF	46294	31	1(1)	1(1)	0.11	Canis lupus familiaris	961 5	CASP4	3	2

Figure S1: Domain organisation of the dog caspase- 1/4/11 hybrid protein which is constitutively expressed in dog DH82 cells, related to Figure 1.

(A) Comparison of the caspase-1 gene subfamily locus in humans and other mammals (from Eckhart et al., 2008). Asterisks indicate the presence of mutations that prevent the expression of a catalytically active caspase. **(B)** Origin of the canine hybrid caspase-1/-4 gene and its two spliced variants, caspase-1/-4/a and caspase-1/-4/b (from Eckhart et al., 2008). Caspase-1/-4/a translates to a protein consisting of a CARD domain similar to human and murine caspase-1 and a caspase domain similar to human caspase-4/-5 and murine caspase-11. Caspase-1/-4/b translates to a protein consisting of two tandem CARD domains, the first showing high homology to the CARD domain of human and murine caspase-1 and the second with the CARD domain of human caspase-4/-5 and murine caspase-11, and a caspase domain with high similarity to the caspase domain of human caspase-4/-5 and murine caspase-11. Boxes represent exons with their numbers shown. **(C)** Ensembl gene build annotation of the hybrid gene (ENSCAFG00000014860) shows the predicted protein coding exons. The identified catalytic residue is present in both transcripts in exon 6. **(D)** Full protein extracts from non-stimulated (medium only) and LPS-primed WT dog DH82 cell lysates were analysed via SDS-PAGE gel electrophoresis and gels stained with Coomassie colloidal stain. Bands between the 32 kDa and 58 kDa molecular weight markers (corresponding to the predicted size of the hybrid protein, area marked with red rectangles) were excised and submitted for mass spectrometry analysis. **(E)** Mass spectrometry analysis showed expression of the hybrid gene in non-stimulated and LPS-treated samples.

A

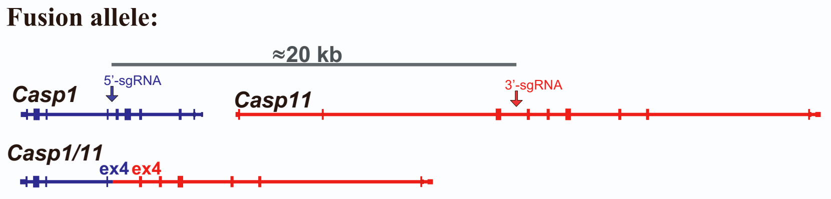

B

```
(CLUSTAL O(1.2.4) multiple sequence alignment)

FUSION      MADKILRAKRKQFINSVSIGTINGLLDELLEKRVLNQEEMDKIKLANITAMDKARDLCDH  60
DOG         MADKVLKDKRRLFVRSVDMGTINGLLDELFEKRVLNHEEMERVRCAHSTVMDQARVLIDS  60
            ****:*: **: *:.**.:***********:******:***:::: *: *.**:** * *

FUSION      VSKKGPQASQIFITYICNEDCYLAGILELQSAPSAETFVATEDSKGGHPSSSETKEE-QN  119
DOG         VLRKGPNACQIFISNICNEDIHLAQTLGLSSGSPSGNDHTKLDSQVEVPSLPAFVENMPG  120
            * :***:*.****: ***** :**  * *.*.  : .  :. **:    **     *:  .

                                 Casp1 ex.4 ↓ Casp4 ex.4
FUSION      KEDGTFPGLTGTLKFCPLEKAQKLWKENPSEIYPIKEANGRTRKALIICNTEFKHLSLRY  179
DOG         PTIPDSEESTDTLKLCPPETFVKMYKEKAEEIYPIKERKDRTRLALIICNIEFDHLSTRD  180
                     *.***:** *.   *::**: .******* :.*** ****** **.*** *

FUSION      GANFDIIGMKGLLEDLGYDVVVKEELTAEGMESEMKDFAALSEHQTSDSTFLVLMSHGTL  239
DOG         GAELDIAGMESLLEGLGYSVVVKRKLTAKGMESVLREFAARPEHKSSDSTFLVLMSHGIL  240
            **::** **:.***.***.****.:***:**** :::***  **::*********** *

FUSION      HGICGTMHSEKTPDVLQYDTIYQIFNNCHCPGLRDKPKVIIVQACRGGNSGEMWIRESSK  299
DOG         NGICGTAHSVENPDVLAYDTIFQIFNNRHCLNLKDKPKVIIIQACRGENPGELWVSDSPK  300
            :***** ** :.**** ****:***** ** .*:*******:***** * **:*: :* *

FUSION      PQLCRGVDLPRNMEADAVKLSHVEKDFIAFYSTTPHHLSYRDKTGGSYFITRLISCFRKH  359
DOG         ASTDSWTHQPLMLKSDAIHKVHVEKDFIAFCSSTPHNVSWRHITKGSLFIAQLITCFQKY  360
             .    .. *  :::**::  ********* *:***::*:*. * ** **::**:**:*:

FUSION      ACSCHLFDIFLKVQQSFEKASIHSQMPTIDRATLTRYFYLFPGN    403
DOG         SWCCHLEGVFRKVQQSFEKPDVKAQMPTIERVSMTRYFYLFPGN    404
            : .*** .:* ******** .:::*****:*.::**********
```

C

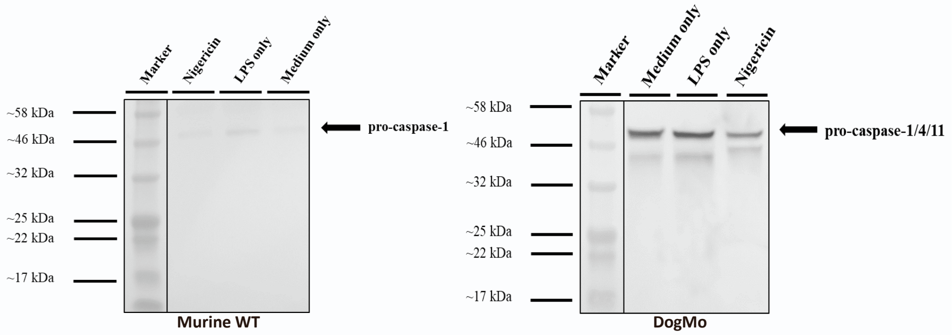

D

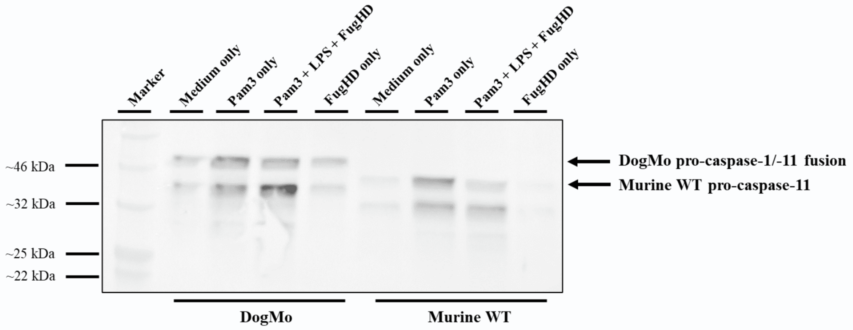

Figure S2

Figure S2: Strategy for the generation of the DogMo mouse and detection of the hybrid caspase-1/-4 protein in DogMo BMDM during canonical and non-canonical inflammasome activation, related to STAR Methods and Figure 1.

(A) To generate a mouse allele corresponding to the dog caspase gene fusion, a CRISPR strategy with two sgRNAs was used to generate a 20,524 bp deletion (GRCm38/mm10 chr9:5,302,869-5,323,392). The 5' sgRNA (binding to chr9:5,302,865-5,302,884 reverse strand) is located in Casp1 intron 4 and the 3' sgRNA (binding to chr9:5,323,374-5,323,393) is located in Casp4 intron 3. **(B)** The deletion created an in-frame fusion between Casp1 exons 1-4 and Casp4 exons 4-9, resulting in a fusion protein similar to the dog fusion protein. **(C)** Mouse WT and DogMo BMDM were primed with LPS (200 ng/ml for 3 hours) followed by stimulation with nigericin (20 µM for 1 hour). Caspase-1 and the hybrid caspase-1/-4 protein expression was confirmed by western blots of WT and DogMo cell lysates, respectively. **(D)** Mouse WT and DogMo BMDM were primed with Pam3CSK4 (10 µg/ml for 4 hours) then stimulated with either LPS (5 µg/ml) alone or in conjunction with Fugene HD transfection reagent. Caspase-11 and the hybrid caspase-1/-4 protein expression was confirmed by western blots of cell lysates. Data have been generated from one single experiment in **C-D**.

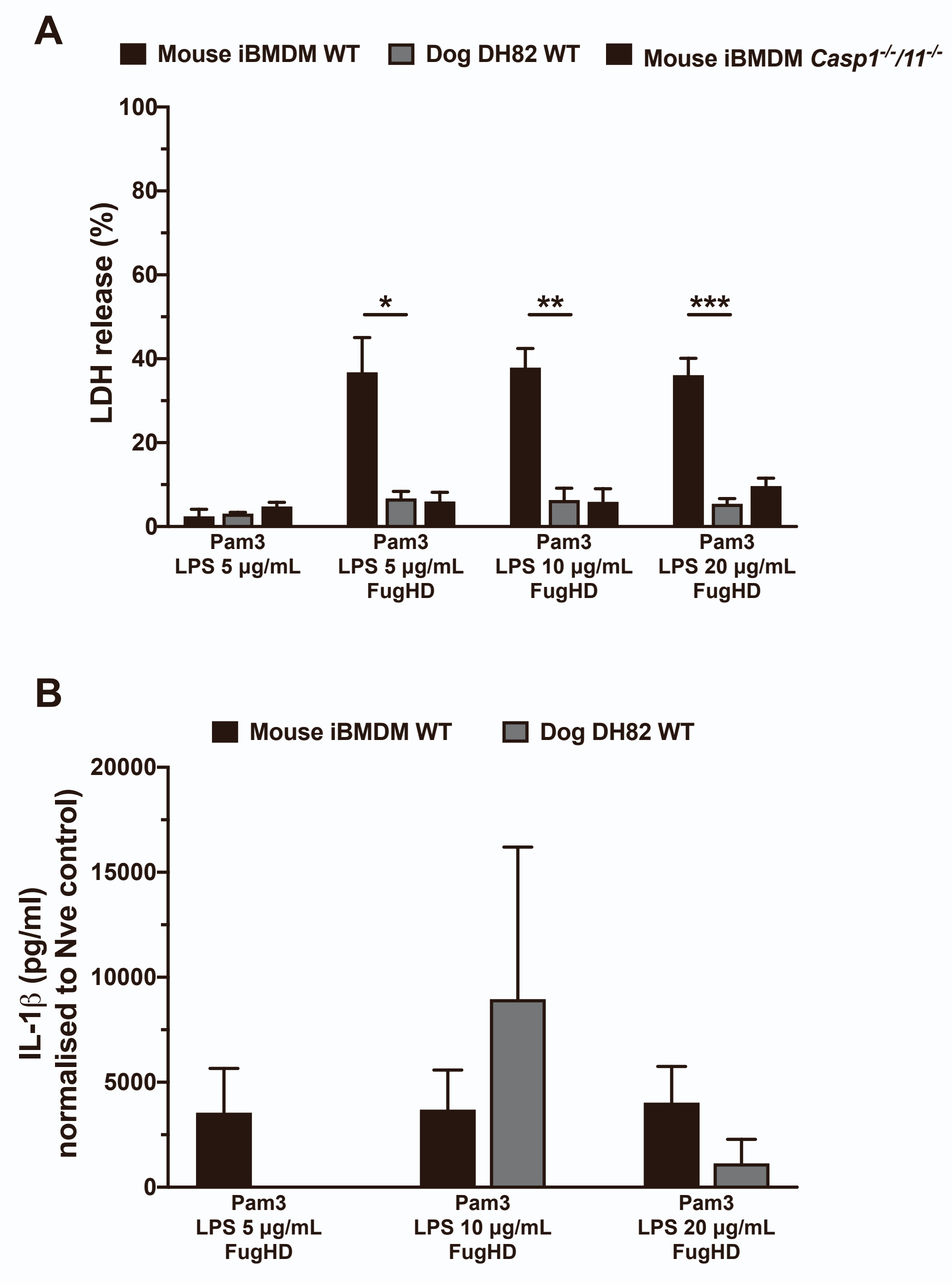

Figure S3: The Carnivora hybrid caspase-1/-4 protein does not mediate non-canonical inflammasome activation, related to Figure 2.

Mouse WT iBMDM, mouse *Casp1*$^{-/-}$*/11*$^{-/-}$ iBMDM and dog WT DH82 cells were primed with Pam3CSK4 (10 μg/ml for 4 hours) and then stimulated with either LPS (5 μg/ml) alone or in conjunction with Fugene HD transfection reagent. **(A)** Percentage of cell lysis induced was determined by measuring LDH release into the supernatant while **(B)** IL-1β levels were measured in the supernatant via ELISA. Data are shown as mean ± s.e.m. and are pooled from three independent experiments. Statistical significance in **A** was calculated by an one-way ANOVA for each LPS concentration individually followed by Tukey's multiple comparison test: *$p<0.05$, **$p<0.01$, ***$p<0.001$.

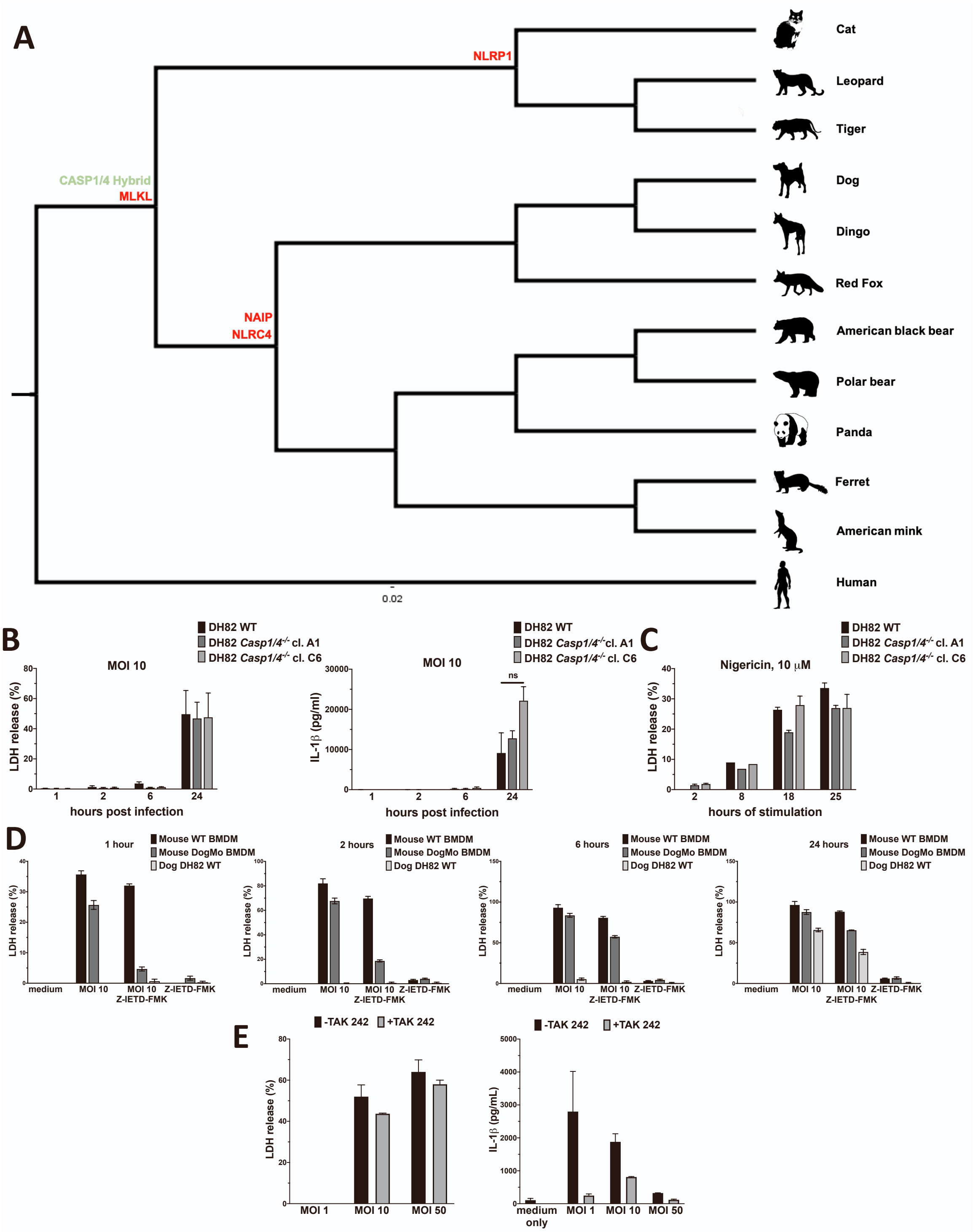
A
NLRP1
CASP1/4 Hybrid
MLKL
NAIP
NLRC4
Cat
Leopard
Tiger
Dog
Dingo
Red Fox
American black bear
Polar bear
Panda
Ferret
American mink
Human
0.02
B
MOI 10
LDH release (%)
hours post infection
DH82 WT
DH82 Casp1/4-/- cl. A1
DH82 Casp1/4-/- cl. C6
IL-1β (pg/ml)
ns
C
Nigericin, 10 μM
hours of stimulation
D
1 hour
2 hours
6 hours
24 hours
Mouse WT BMDM
Mouse DogMo BMDM
Dog DH82 WT
medium
MOI 10
Z-IETD-FMK
E
-TAK 242
+TAK 242
MOI 1
MOI 50
IL-1β (pg/mL)
medium only

Figure S4. The delayed cell death seen in dog cells is driven by caspase-8 rather than the hybrid caspase-1/-4 protein, related to Figure 4.

(A) Species tree of the order Carnivora available on the Ensembl database showing gain and loss of genes. Gene loss is represented by gene names labelled in red, while gene gain (or substitution) is represented by gene names labelled in green. **(B-C)** Dog WT DH82 (electroporated only) and cells from two individual DH82 *Casp1/4*$^{-/-}$ clones Caspase-1/-4 knock-out clones (CRISPR-generated) were infected with *S.* Typhimurium at an MOI of 10 or primed with LPS (200 ng/mL for 3 hours) and stimulated with nigericin (10 μM for 1 hour). **(B)** Levels of LDH and IL-1β released in the supernatant in response to infection were measured via a colorimetric assay and ELISA, respectively. **(C)** Levels of LDH released in the supernatant in response to LPS/nigericin treatment were measured as in **B**. **(D)** Mouse WT BMDM, mouse DogMo BMDM and dog WT DH82 cells were infected with *S.* Typhimurium MOI of 10 in the presence of selective caspase-8 inhibitor (Z-IETD-FMK, 10 μM). Levels of LDH released in the supernatant were measured at 1, 2, 6 and 24 hours post-infection via a colorimetric assay. **(E)** Dog WT DH82 cells were infected with *S.* Typhimurium at an MOI of 1, 10 and 50 in the presence of TLR4 selective inhibitor TAK 242 (1 μM). Levels of LDH and IL-1β were measured in the supernatant 24 hours after infection via a colorimetric assay and ELISA, respectively. Data are shown as mean ± s.e.m. Data shown are pooled from three independent experiments in **B,** from one experiment representative of two with similar results in **C** and from one single experiment in **D** and **E**. Statistical significance in **B** was calculated by an one-way ANOVA for each time point individually followed by Tukey's multiple comparison test: ns=not significant.

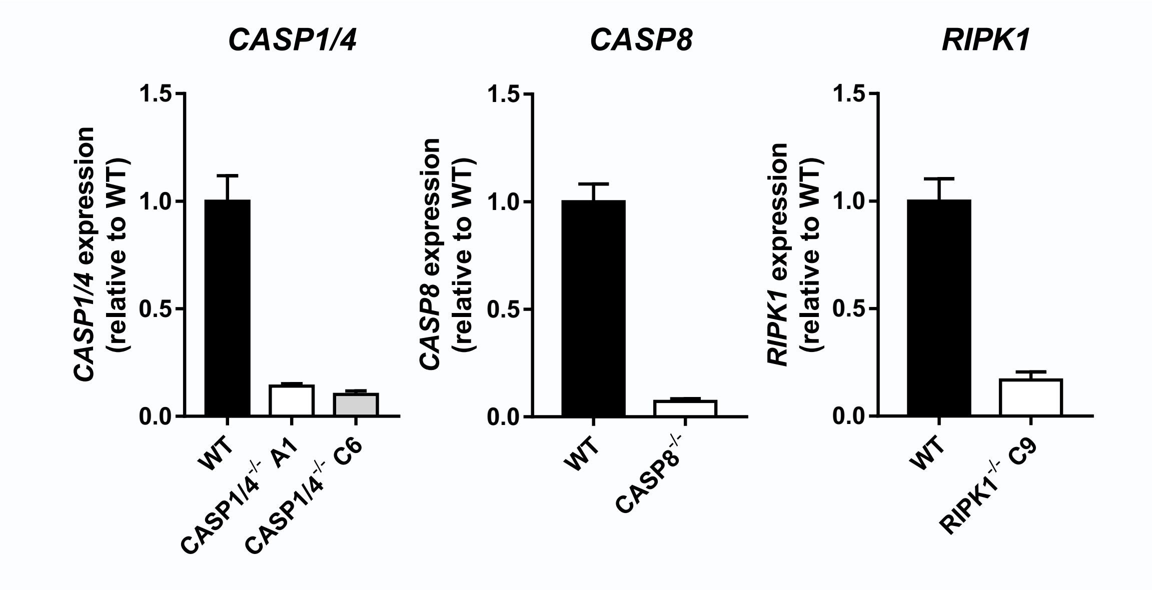

Figure S5: Quantitative PCR validation of gene disruption in CRISPR/Cas9 edited DH82 cells, related to STAR Methods.

Quantitative PCR analysis of CASP-1/4, CASP8, and RIPK1 expression in dog WT DH82, *casp1/4*$^{-/-}$ clones A1 and C6, *casp8*$^{-/-}$ bulk-edited and *ripk1*$^{-/-}$ clone C9. Data are shown as mean ± 95% confidence level from three technical replicates and are representative of two independent experiments.

Table S1. Details of canine patients used as donors for isolating blood PBMCs, related to STAR Methods, Figure 1 and 2.

Identifier	Breed	Age	Gender	Reason for blood test
1379 2901	Whippet	9 years	Male	Chemo monitoring
1379 3501	Retriever	12 years	Male	Monitoring of treatment for anal sac adenocarcinoma
1379 4801	Cocker Spaniel	Unknown	Male	Monitoring of immune-mediated haemolytic anaemia
1379 5301	Staffordshire Bull Terrier	11 years	Female	Chemo monitoring
1379 5401	Labradoodle	12 years	Female	Screening
1379 6201	Greyhound	6 years	Female	Chemo monitoring
1386 0901	Pug	5 years	Male	Protein-losing enteropathy
1386 1301	Cacapoo	4 years	Male	Immune Mediated Thrombocytopenia re-check
1386 3601	Staffordshire Bull Terrier	8 years	Female	Screening

Table S2. Primers used for quantitative PCR analysis of CASP-1/4, CASP8, and RIPK1 gene expression, related to STAR Methods.

Gene	Forward primer (5'-3')	Reverse primer (5'-3')
HPRT1	CCCCAGCGTCGTGATTAGTG	AACACTTTTTCCAAATCCTCAGCG
GAPDH	CCCTGAGCTGAACGGGAAG	CTCCGATGCCTGCTTCACTAC
CASP1/4	CTTCGGAAAGGGCCAAATGC	TGTTTTCCACGAAGGCTGGT
CASP8	TGACTTTCTGCTGGGGATGG	TGTATATACCATGTCCCCTCCAT
RIPK1	GGCCAACATTTGCTGGCATT	TGACCTGCTTGGAGGTGTTG
